# The Beneficial Effects of Inoculation with Selected Nodule-Associated PGPR on White Lupin Are Comparable to Those of Inoculation with Symbiotic Rhizobia

**DOI:** 10.3390/plants12244109

**Published:** 2023-12-08

**Authors:** Abdelhakim Msaddak, Miguel A. Quiñones, Mohamed Mars, José J. Pueyo

**Affiliations:** 1 Department of Soil, Plant and Environmental Quality, Institute of Agricultural Sciences, ICA-CSIC, 28006 Madrid, Spain; ma.quinones@csic.es; 2 Laboratory of Biodiversity and Valorization of Arid Areas Bioresources, BVBAA, Faculty of Sciences, University of Gabès, Erriadh, Zrig, Gabès 6072, Tunisia; marsmohamed@yahoo.fr

**Keywords:** lupin, *Lupinus albus*, rhizobia, *Bradyrhizobium*, plant growth-promoting rhizobacteria (PGPR), nodule, endophytes

## Abstract

Nodule endophytes and associated bacteria are non-symbiotic bacteria that colonize legume nodules. They accompany nodulating rhizobia and can form beneficial associations, as some of them are plant growth-promoting rhizobacteria (PGPR) that are able to promote germination and plant growth and increase tolerance to biotic and abiotic stress. White lupin (*Lupinus albus*) is a legume crop that is gaining relevance as a suitable alternative to soybean as a plant protein source. Eleven nodule-associated bacteria were isolated from white lupin nodules grown in a Tunisian soil. They belonged to the genera *Rhizobium*, *Ensifer*, *Pseudomonas* and *Bacillus*. Their plant growth-promoting (PGP) and enzymatic activities were tested in vitro. Strains *Pseudomonas* sp., L1 and L12, displayed most PGP activities tested, and were selected for *in planta* assays. Inoculation with strains L1 or L12 increased seed germination and had the same positive effects on all plant growth parameters as did inoculation with symbiotic *Bradyrhizobium canariense*, with no significant differences among treatments. Inoculation with efficient nitrogen-fixing rhizobia must compete with rhizobia present in the soil that sometimes nodulate efficiently but fix nitrogen poorly, leading to a low response to inoculation. In such cases, inoculation with highly effective PGPR might represent a feasible alternative to boost crop productivity.

## 1. Introduction

White lupin (*Lupinus albus*) serves predominantly as a protein source in animal and human diet owing to the significant protein content found in its seeds. There is a growing trend of integrating it into contemporary agricultural and food systems to enhance the production of superior plant-based proteins [1]. Lupin has a history of being utilized as a medicinal plant to address a range of health conditions [2]. Lupin seeds contain proteins, fatty acids, amino acids, alkaloids, minerals, and dietary fiber, and separating these components allows for the extraction of substances with proven effectivity in combating diabetes or cancer, reducing inflammation, lowering blood pressure, and acting as antioxidants [3,4]. White lupin plants have also been described to be effective in heavy metal phytoremediation and the recovery of degraded soils [5]. *Lupinus albus* demonstrates a notable capacity for enduring high levels of heavy metals, displaying resilience in stressful environments. Reports indicate that white lupin, when inoculated with a mercury-resistant *Bradyrhizobium* strain, exhibits heightened tolerance to this heavy metal [6]. Phosphorus and nitrogen, along with potassium, stand as fundamental elements for sustaining crop productivity. They play a pivotal role in supporting crop growth. In phosphate-deficient soils, white lupin possesses the ability to alter its root structure, forming what is known as cluster roots [7] which exhibit elevated production and release of acidic organic compounds, phosphatases, flavonoids, and protons. These alterations result in the solubilization of inaccessible phosphorus in the soil. Therefore, lupin reduces the need for both N and P fertilizers, and represents a choice crop for nutrient-poor soils [7]. Furthermore, these described mechanisms render white lupin a suitably adapted plant for acidic soil conditions impacted by aluminum toxicity [8]. Its capacity to thrive in environments with low nutrient content, acidity, or moderate pollution levels, as well as in both dry and cold climates, allows this legume to grow in a wide range of edaphoclimatic conditions [1,9].

Besides symbiotic rhizobia, which are soil bacteria able to form root nodules where they are able to fix atmospheric nitrogen [10], other bacteria can colonize legume nodules. They are known as non-rhizobial nodule-associated bacteria (NAB) [11], non-rhizobial endophytes (NRE) [12], or simply nodule endophytes [13]. These non-nodulating bacteria accompany rhizobia and can form beneficial associations with them. The term PGPR as an acronym for plant growth-promoting rhizobacteria was first used by Kloepper et al. [14], and ever since, PGPR have been increasingly seen as a way of complementing traditional inputs in agricultural systems [14,15], as interactions between plants and PGPR have demonstrated advantages in enhancing plant development and resilience against stress.

Some endophytic and rhizospheric bacteria have different PGPR traits and possess plant growth-promoting (PGP) properties. PGPR aid plants through both direct and indirect processes, including acquiring nutrients like phosphorus, nitrogen, and iron, producing phytohormones, and regulating ethylene levels [16,17]. Atmospheric N fixation [18], P mobilization/solubilization [19] and siderophores production [20,21] are the major beneficial mechanisms involved in PGPR-aided nitrogen, phosphate and iron acquisition by plants. Moreover, PGPR have the capability to produce and release plant hormones like auxins, particularly indole-3-acetic acid (IAA) involved in plant and root growth and status, stimulating root elongation and improving nutrient acquisition [22,23]. PGPR can reduce the stress level in plants by production of the enzyme amino-cyclopropane carboxylic acid (ACC) deaminase that breaks the ethylene precursor (ACC) decreasing the levels of this phytohormone. They also improve the acquisition of nutrients [24,25] and increase nodulation [26]. PGPR can affiliate to several genera including *Acinetobacter*, *Bacillus*, *Pseudomonas* or *Variovorax* among others [27,28,29]. Several reports have shown the PGPR characteristics and positive effects of nodule-associated bacteria in *L. luteus*, *L. angustifolius* and other legume species such as *Medicago sativa* or *Pisum sativum* [30,31]. In addition, rhizobial strains belonging to genera that can nodulate lupin and/or other legumes, including *Rhizobium*, *Neorhizobium*, *Agrobacterium* or *Burkholderia*, have been described as lupin nodule endosymbionts or associate bacteria [32].

This study aimed to isolate and characterize bacteria associated with *L. albus* nodules while assessing their plant growth-promoting (PGP) characteristics. This included examining traits like ACC deaminase activity, siderophore and IAA production, as well as their ability to fix nitrogen and solubilize phosphate and zinc. Evaluation of the effects of selected nodule-associated bacteria on plant growth promotion was tested in *L. albus* plants by biopriming seeds with individual PGPR isolates or in consortia with lupin microsymbiont *Bradyrhizobium canariense* in order to assess their ability to promote lupin growth, to compare their effects with those of rhizobial inoculation, and their possible synergistic effects with *B. canariense*.

## 2. Results

### 2.1. Isolation and Molecular Characterization of Nodule-Associated Bacteria

Twenty-two isolates (L1 to L22) were initially obtained from root nodules of *L. albus* grown in a soil from Ghannouch, Southern Tunisia, based on colony color and morphology. Analysis of RAPD-PCR fingerprints of the initial 22 isolates allowed the identification of 11 different patterns or profiles. One representative of each different pattern was selected for gene sequencing in order to determine what genera and species the strains belonged to.

**Figure 1:**
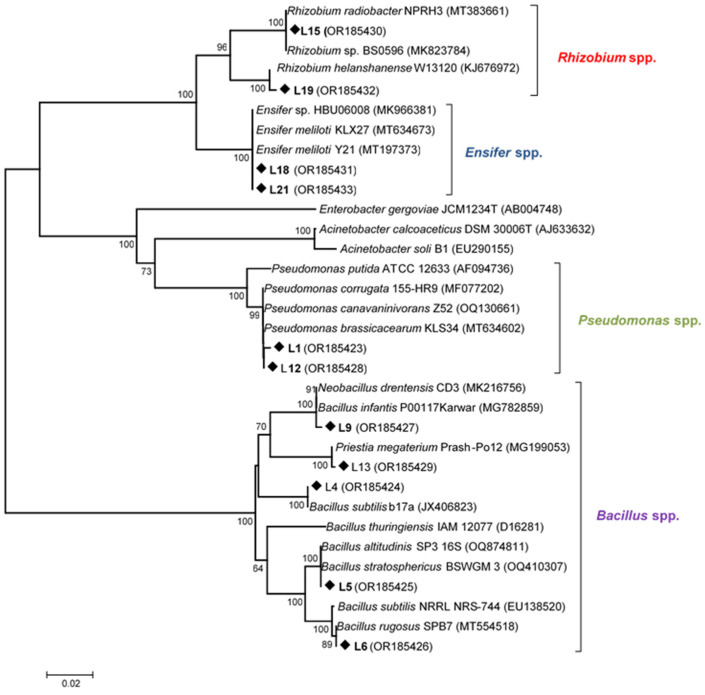
Neighbor-joining phylogenetic analysis of the 16S rRNA gene sequences of the bacterial isolates (◆) and reference strains. GenBank accession numbers are in parentheses. Bootstrap values greater than 50% are indicated.

Near full-length 16S rRNA genes of the 11 strains were amplified and sequenced by PCR. BLASTN search results showed that all sequences clustered with fast growing bacteria genera. A neighbor-joining phylogenetic tree was performed based on the 16S rRNA gene sequences of the strains and the corresponding sequences from type strains. The phylogeny based on 16S rRNA gene sequences indicated that the 11 selected isolates belonged to four different genera (Figure 1). The phylogenetic analysis revealed that the isolates presented 95–100% identity with available sequences in GenBank. Two strains were identified as *Pseudomonas* sp. (L1, L12). The rest of the isolates affiliated to the genera *Bacillus* (L4, L5, L6, L9 and L13), *Ensifer* (L18, L21) and *Rhizobium* (L15, L19). L15 showed 100% identity with *R. radiobacter*. L19 had high sequence identity with *R. helanshanense*. L18 and L21 16S rRNA gene sequences were 100% identical to *E. meliloti*, L1 and L12 had high identity with *P. brassicacearum*. L4, L5, L6, L9 and L13 presented high identity with *B. subtilis* b17a, *B. stratosphericus* BSWGM 3, *B. rugosus* SPB7, *B. infantis* P00117Karwar and *B. megaterium* Prash-Po12, respectively (Figure 1).

### 2.2. PGP Properties and Enzymatic Activities of the Isolates

The results of the tested activities are summarized in Table 1. Eight of the isolates showed the ability to grow in a medium devoid of nitrogen, indicating their ability to assimilate atmospheric nitrogen. Five lupin nodule-associated strains were able to solubilize phosphate in NBRIP medium, as they were able to grow and make a clear transparent halo zone surrounding the bacterial colony. The production of IAA was tested in growth medium supplemented with L-tryptophan. Seven isolates were able to produce IAA. Five strains were able to produce siderophores. Siderophore production can provide essential metals such as zinc and iron [33]. However, none of the tested strains was able to solubilize Zn.

The level of ACC deaminase activity was measured through the production of α-ketobutyrate, a result of the deamination process of ACC catalysis. Only two isolates, classified as *Pseudomonas* sp., L1 and L12, displayed ACC deaminase activity. The presence of enzymatic activities related to organic matter degradation in the selected strains was also analyzed. Seven strains presented lipase activity. Strains L1, L12, L18 and L21 showed cellulase activity, and all strains were negative for pectinase. The two *Pseudomonas* sp. strains, L1 and L12, displayed most PGP activities tested, plus enzymatic activities lipase and cellulase, and thus they were selected for the inoculation assays.

### 2.3. pH and Temperature Growth Range and Tolerance to Salt and Heavy Metals

The strains were additionally evaluated using certain observable traits like their growth range across varying pH levels and temperatures and their tolerance to NaCl and heavy metals. All isolates were phenotypically classified as fast growers, with 24–48 h of incubation time to see colonies. The best conditions for optimal growth across all strains were observed at a temperature of 28 °C and a pH of 7. While the isolates displayed a broad range of characteristics, each of them demonstrated the ability to proliferate within a temperature range from 25 to 40 °C and a pH range from 4 to 12 (except for L9; Table 2). In general, most isolates showed a certain tolerance to NaCl from 340 to 1200 mM. Strains L5, L6 and L13, affiliated to the genus *Bacillus*, were able to grow in 1200 mM NaCl (Table 2). L9 was very sensitive to acidity, alkalinity and salt, and was negative for all PGP activities (Table 2).

The tolerance of the eleven rhizobial strains to six different metals was tested, and the maximum tolerable concentrations (MTC) for each metal and strain are shown in Table 2. According to the definition of metal tolerance for eubacteria [34], strains that are not inhibited by 1 mM Cr, Co, Ni, Cu, Zn, Ag, Cd and Pb (MIC > 1 mM) are considered tolerant. All tested strains were tolerant to Pb, Cu and Ni but sensitive to Cd, Zn and Cr (Table 2).

### 2.4. Effects of Selected PGPR Strains on Germination, Growth, and Nodulation of L. albus Plants

**Figure 2:**
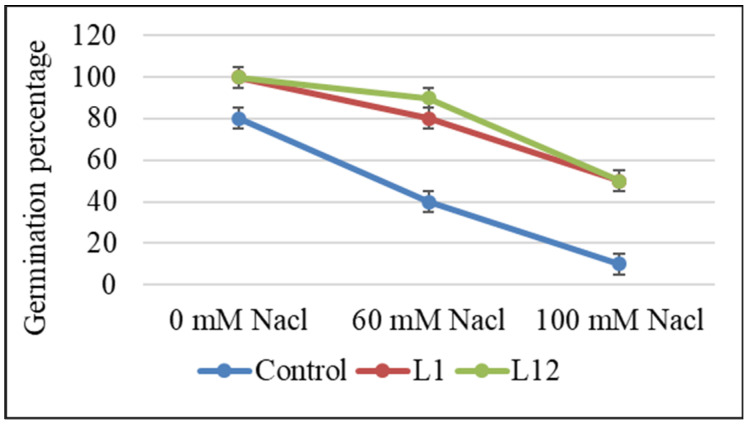
In vitro effects of selected PGPR strains (L1 and L12) on the percentage of *L. albus* seed germination at different salt concentrations. Data presented are means ± SD (*n* = 30).

Strains *Pseudomonas* sp. L1 and L12 were used to inoculate *L. albus* seeds to test their effect on germination. Figure 2 shows that the seeds subjected to inoculation exhibited a greater germination percentage compared to control seeds that were not inoculated, reaching 100% germination versus 80% for non-inoculated ones. They also increased germination relative to control seeds in the presence of 60 and 100 mM NaCl, indicating a certain protection against salinity stress.

**Figure 3:**
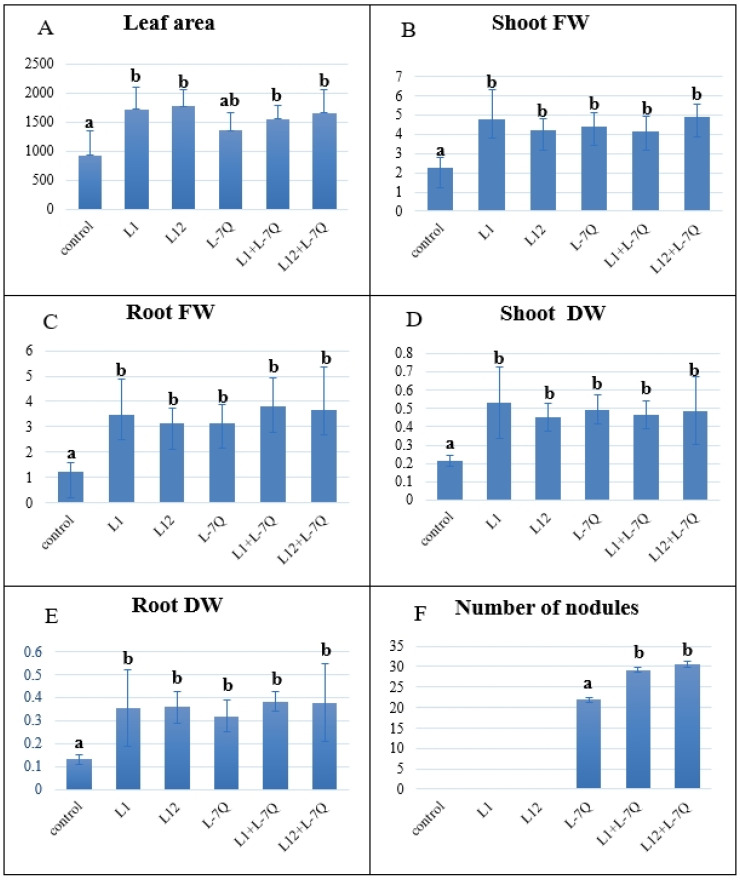
Effects of inoculation of *L. albus* plants with nodule-associated *Pseudomonas* sp. L1 and L12, *Bradyrhizobium canariense* L-7Q and consortia thereof. (**A**) Leaf area (mm^2^); (**B**) shoot fresh weight (g); (**C**) root fresh weight (g); (**D**) shoot dry weight (g); (**E**) root dry weight (g); (**F**) number of nodules. Data presented are means ± SD (*n* = 6). Different letters denote significant differences between treatments.

The growth of plants assessed by various growth metrics, encompassing leaf area, and shoot and root fresh and dry weights, was significantly increased by inoculation with either bacteria or bacterial combination when compared to control plants (Figure 3A–E). No significant differences among the inoculation treatments were observed. Regarding nodulation, control plants and plants inoculated with PGPR L1 and L2 presented no nodules, while symbiotic *B. canariense* L-7Q efficiently nodulated *L. albus* plants. The number of nodules in plants that were co-inoculated with L-7Q and either L1 or L12 was significantly higher than in those inoculated with L-7Q alone (Figure 3F).

## 3. Discussion

Twenty-two nodule-associated bacteria were isolated from nodules of *L. albus* grown in a soil from Ghannouch, Southern Tunisia. The BoxA1 random amplified polymorphic DNA PCR technique (RAPD-PCR) has proven useful to assess rhizobial diversity [13,35,36,37,38] and allowed the identification of eleven strains that presented different band patterns. According to their 16S rRNA gene sequence analysis, the eleven isolates were shown to belong to the genera *Rhizobium*, *Ensifer*, *Pseudomonas* and *Bacillus.*

Two isolates belonged to the genus *Rhizobium*, and one of them presented 100% identity with *R. radiobacter*, also known as *Agrobacterium tumefaciens.* In fact, Young et al. [39] proposed the inclusion of all *Agrobacterium* species under the genus *Rhizobium. Agrobacterium* sp. (now *Rhizobium* sp.) strains appear to be dominant and account for over half of the total isolates obtained from *L. albus* nodules in Tunisia [32]. In general, *Agrobacterium*/*Rhizobium* species are ubiquitous in soil and plant habitats [40]. Numerous research studies document the extraction of *Agrobacterium*/*Rhizobium* strains from nodules of legumes hosting symbiotic rhizobia [41,42]. Two other isolates belonged to the genus *Ensifer*. There are very few reports that describe *Ensifer* as a PGPR, but *E. adhaerens* has been described as an endophytic PGPR isolated from nodules of *Vigna unguiculata* [43]. Isolates *Pseudomonas* sp. L1 and L12 were selected for inoculation assays because they were positive for most PGP activities tested. There is abundant literature describing *Pseudomonas* sp. bacteria as effective PGPR with a vast variety of crops. *Pseudomonas* strains have been isolated from nodules of different legumes, including lupin [31], and inoculation of selected *Pseudomonas* strains has been reported to promote *L. albus* growth and biomass [31,44]. Our results showed that five out of the eleven PGPR strains were identified as *Bacillus* sp. *Bacillus* is one of the most abundant nodule endophytes and rhizobacteria [45,46]. *Bacillus* PGPR have been reported to improve the yields of various crops by stimulating plant growth and improving nutrient supply [47,48]. It is noteworthy that one of our *Bacillus* isolates (L9) did not present any of the PGP activities tested, and it was especially sensitive to pH, temperature, salt and heavy metals, which highlights the fact that not all nodule endophytic *Bacillus* strains are necessarily PGPR.

Inoculation of *L. albus* seeds with the selected nodule-associated bacteria resulted in an increased germination percentage in the presence and absence of salt. IAA-producing bacteria have been described to enhance plant seed germination [49,50]. However, Miransari and Smith [51] showed that auxin should not be considered the only necessary hormone for seed germination. The possibility cannot be excluded that L1 and L12 could also secrete other compounds, such as gibberellins and/or cytokinins, which are known to enhance seed germination under salt stress conditions [51]. Salt-tolerant isolates L1 and L12 might facilitate lupin seed germination and possibly plant growth in soils facing salinity stress.

The selected *Pseudomonas* sp. L1 and L12 were able to grow in a nitrogen-free minimal medium, suggesting their potential for nitrogen fixation, which might further augment the nitrogen-fixing capacity within the nodules [52]. Phosphate solubilization is another important parameter of PGPR. Our results align with numerous published studies that document the ability of *Bacillus* or *Pseudomonas* species to solubilize phosphate [53,54]. Phosphate solubilization would allow strains L1 and L12 to provide phosphorous to plants growing in phosphate-deficient soils. The production of siderophores stands as a crucial property for plant growth promotion in nutrient-deficient soils due to their iron-binding capacity. These compounds create complexes that roots can absorb and assimilate, aiding in nutrient uptake [55]. Moreover, the generation of siderophores is interconnected with biotic control as it involves competition with phytopathogens for iron resources [56]. Phosphate solubilization and siderophore production have been reported by several authors as fundamental for plant growth, development and resilience [29,57,58]. Concerning IAA production, which is involved in plant root elongation and in the formation of lateral roots and root hairs [59], many of the tested strains, including L1 and L12, were able to produce different hormone levels. This promotion of the root system improves nutrient and water uptake efficiency. ACC deaminase activity, detected only in *Pseudomonas* sp. L1 and L12, enables bacteria to control the level of ethylene by breaking down ACC, its precursor [60,61]. Thus, bacteria promote plant growth under stress conditions [62]. The synthesis of lytic enzymes, like pectinases and lipases, which target the walls of plant pathogens, contributes as an attribute that increases plant resilience against biotic stress [60]. Cellulase activity is important in establishing symbiosis as it degrades the plant cell wall and facilitates the entry of rhizobia in roots [63]. Strains L1 and L12 displayed cellulase and lipase enzymatic activities. This could be one of the reasons why plants inoculated with the *Pseudomonas-Bradyrhizobium* consortia presented a higher number of nodules than when inoculated with *Bradyrhizobium* alone.

Inoculation with *Pseudomonas* sp. strains L1 or L12 had the same positive effects on all plant growth parameters as did inoculation with *B. canariense*, with no significant differences among the treatments. Co-inoculation, though, did not improve plant growth as compared to inoculation with the microsymbiont alone. The good scores of L1 and L12 in most PGP properties allowed them to promote growth in lupin in a comparable manner to symbiotically efficient *B. canariense* L-7Q. L1 and L12 were capable of providing the necessary nitrogen for optimal growth. Additionally, their IAA and ACC deaminase production might have promoted root growth in a coordinated manner.

*Pseudomonas* sp. strains, L1 and L12, demonstrate exceptional efficacy as PGPR, suggesting their potential use in creating eco-friendly biofertilizers aimed at enhancing the productivity of white lupin. Moreover, in general, PGPR are effective with different crops, and both L1 and L12 presented the necessary PGPR characteristics to improve plant growth in a variety of soils as they were able to grow in a wide pH and temperature range, plus they were tolerant to salt stress and to several heavy metals. The fact that they provided the same effects as efficient symbiotic bacteria can be of special interest when resident soil rhizobia are not highly effective in nitrogen fixation, which is relatively frequent for some legumes and soils, especially common bean [64]. Efficient nitrogen-fixing rhizobial inocula must compete with rhizobia present in the soil that nodulate efficiently but fix nitrogen poorly. This competition might lead to a low response to inoculation [64]. In such cases, inoculation with powerful PGPR, such as strains L1 and L12, might represent a feasible advantageous alternative to boost crop yield.

## 4. Materials and Methods

### 4.1. Soil Samples Collection and Characterization

Soil samples were collected in Ghannouch, Southern Tunisia (33°93′22.37″ N, 10°07′91.56″ E), in January 2023. After removing the superficial layer, soil samples (15–20 cm deep) were collected and deposited into plastic bags. The soil texture was 50.4% sand, 7.2% clay and 42.4% silt. The soil pH was 8.1 and its electric conductivity 11.95 μS cm^−1^. The samples were immediately transported to the laboratory, sieved and stored at 4 °C until use.

### 4.2. Isolation and Bacterial Growth

Bacterial strains were obtained from the root nodules of *L. albus* trap plants found in the gathered soil. The roots were rinsed thoroughly with distilled water. Surface sterilization of the nodules began with a 1 min treatment using 95% ethanol, followed by exposure to 25% sodium hypochlorite (approximately 10% active chlorine) for 3 min. Afterwards, the nodules underwent multiple rinses with sterile water and subsequently crushed onto sterile plates and streaked onto YMB agar medium. These plates were then kept in incubation at 28 °C for 72 h [65]. The purity of the isolates was ensured by picking and re-streaking individual colonies. Bacteria with different colony morphology were selected for further identification. Bacterial samples were preserved either on agar slants at 4 °C or frozen in liquid growth medium with 50% glycerol at −80 °C for long-term storage.

### 4.3. Analysis of Diversity and Identification of Isolates

#### 4.3.1. DNA Isolation and PCR Amplification

Bacterial genomic DNA was extracted by the alkaline lysis method [65]. Fresh bacterial colonies were placed in 20 µL of a lysis solution (0.05 M NaOH, 0.25% SDS) and heated to boiling for 15 min. The resulting lysate was diluted in 100 µL sterile distilled water and centrifuged at 13,000× *g* for 10 min. The supernatant was collected for PCR amplification.

The diversity of the isolated bacteria was analyzed by Box-PCR in 25 µL reaction medium containing 1 µL DNA (5–10 ng), 2.5 µL of 10 × PCR buffer with magnesium chloride, 10 mM dNTPs, 10 µM BoxA1R primer, 1 µL DMSO and 1 U Taq DNA polymerase. The box random amplified polymorphic DNA polymerase chain reaction technique (RAPD-PCR) was used to generate genomic fingerprints of the isolates, using the BoxA1R primer (5′-CTACGGCAAGGCGACGCTGACG-3′) [29] and according to the following amplification conditions: initial denaturation at 94 °C (2 min), 35 cycles of denaturation at 94 °C (1 min), annealing at 52 °C (1 min), extension at 65 °C (6.5 min), and final extension at 68 °C (8 min). Electrophoresis was carried out for 2 h in a 1.5% (*w*/*v*) agarose gel at 70 V.

Distinct bacteria from each unique Box-PCR profile were identified through the amplification of the 16S rRNA gene using fD1 (5′-AGA GTT TGA TCC TGG CTC AG-3′) and rP2 (5′-ACG GCT ACC TTG TTA CGA CTT-3′) primers [66]. PCR amplification was carried out and sequenced following procedures already described elsewhere [65]. Unincorporated primers and dNTPs were removed from the PCR products with the PureLink^TM^ Quick PCR Purification Kit (Invitrogen) or, when needed, by gel electrophoresis followed by band purification with the same kit. Sequencing by the Sanger method [67] was performed externally at the Genomics Unit (IPBLN), Institute of Parasitology and Biomedicine López-Neira (CSIC, Granada, Spain).

#### 4.3.2. Phylogenetic Analysis

Sequences were compared with those obtained from GenBank using the BLASTN program (http://www.ncbi.nlm.nih.gov/blast (accessed on 26 October 2023)) and the EzBiocloud Database (https://www.ezbiocloud.net/ (accessed on 26 October 2023)) [68], and they were aligned using CLUSTALW [69]. Phylogenetic analyses were carried out using the MEGA7.0 software [70]. The neighbor-joining statistical methods and the Kimura two-parameter model were used. Phylogenetic trees were boot strapped with 1000 bootstrap replications. Accession numbers of the selected gene sequences were obtained after depositing the nucleotide sequences in the GenBank sequence database.

### 4.4. Plant Growth-Promoting Traits of Selected Nodule-Associated Bacteria

To test bacteria for their in vitro PGP activities, the following five parameters were evaluated: nitrogen fixation, phosphate solubilization, IAA production, ACC-deaminase activity and siderophore production.

#### 4.4.1. Nitrogen Fixation

For detection of nitrogen fixation, the bacteria isolated were plated on nitrogen-free medium (NFB) [71] (*n* = 3 plates). After incubation for 72 h at 28 °C, OD_600_ was adjusted to 0.6. Growth in NFB medium was indicative of bacterial N-fixing activity.

#### 4.4.2. Phosphate Solubilization

NBRIP medium (2.5 g L^−1^ Ca_3_(PO_4_)_2_; 5 g L^−1^ MgCl_2_·6H_2_O; 0.25 g L^−1^ MgSO_4_·7H_2_O; 0.2 g L^−1^ KCl, 0.1 g L^−1^ (NH4)_2_SO_4_, and 15 g L^−1^ agar) supplemented with 10 g L^−1^ glucose (pH 7) was used to check phosphate solubilization [72]. A total of 2 µL drops of exponentially growing bacterial suspension (OD_600_ = 0.6) were spotted onto agar plates. The test was performed in triplicate. The presence of a transparent halo zone surrounding the bacteria was examined after 3 days of incubation at 28 °C and the diameter of the clear halos was measured.

#### 4.4.3. Indole Acetic Acid (IAA) Production

For indolic compound determination, the bacterial isolates were incubated in liquid YM extract medium (5 g L^−1^ peptone, 3 g L^−1^ yeast extract, 3 g L^−1^ malt extract, pH 6.8) with or without tryptophan (1 g L^−1^) at 150 rpm and 28 °C, for 2 days. Auxin production was estimated with the Salkowski reagent (0.5 M FeCl_3_ in 35% HClO_4_ solution) [73]. Flasks were used as biological replicates (*n* = 3), and each one was assayed in triplicate. The tubes were then kept in the dark for 30 min before measuring the optical density at 535 nm. A calibration curve was made with 0, 0.5, 1, 2, 5, 10, 15, 20, 25, 30 mg mL^−1^ of IAA.

#### 4.4.4. 1-Amino-Cyclopropane-1-Carboxylate (ACC) Deaminase Activity

DFS medium was prepared following a previously described protocol [24]. Glucose was filter sterilized and added to the autoclaved medium. To conduct the experiment, bacteria were grown in TY medium for 72 h at 28 °C, 140 rpm. Then, the culture was washed twice with 5 mL of M9 medium and resuspended in 3 mL of M9 medium containing 3 mM ACC. Each strain was tested in triplicate. Culture medium free of bacteria was used as a blank. The analysis involved assessing the reaction by contrasting the absorbance at 540 nm in the samples against an α-ketobutyrate standard curve. To calculate the total protein concentration in toluene-treated cells, a protein calibration curve using bovine serum albumin (BSA) was employed [74]. Ultimately, the measurement of ACC deaminase activity was conducted by assessing the amount of α-ketobutyrate released, expressed as micromoles per milligram of protein per hour. The α-ketobutyrate is produced when the ACC deaminase cleaves the substrate ACC [24,75].

#### 4.4.5. Siderophore Production

The production of siderophores was assessed according to the chrome azurol-S (CAS) agar method [76]. A total of 2 μL drops of pure bacterial culture grown in LB (OD_600_ = 0.6) were inoculated onto agar-CAS plates. Plates were incubated at 28 °C for 3 days. Siderophore production was revealed by the formation of an orange halo around each bacterial colony. Experiments were performed in triplicate (*n* = 3).

#### 4.4.6. Zinc Solubilization Assay

The zinc solubilizing ability of the isolates was determined by spot inoculating the isolates in Tris-minimal medium (6.06 g Tris–HCl; 4.68 g NaCl; 1.49 g KCl; 1.07 g NH_4_Cl; 0.43 g Na_2_SO_4_; 0.2 g MgCl_2_·2H_2_O; 30 mg CaCl_2_·2H_2_O in 1 L distilled sterilized water, pH 7.0) [77] supplemented with 1.5% agar and 0.1% (*w*/*v*) insoluble zinc in the form of zinc sulfate (ZnSO_4_) [78]. The plates were incubated for 14 days at 30 °C and examined for the formation of halos around colonies indicating zinc solubilization.

#### 4.4.7. Lipase Production

LB medium (10 g tryptone, 10 g NaCl, 5 g yeast extract, 15 g agar in 490 mL distilled water) was prepared and 1% Tween-20 was added. A total of 2 µL of each isolate (OD_600_ = 0.6) was spot-inoculated in duplicate. One set of plates underwent incubation at 28 °C for 4 d. Lipase production was perceived by the appearance of a precipitate around the colonies. The experiment was independently replicated three times.

#### 4.4.8. Cellulase Production

The medium used for this test was the same as for lipase production supplemented with 1% carboxymethyl cellulose (CMC) instead of Tween-20. A total of 2 µL of each isolate (OD_600_ = 0.6) was spot-inoculated onto the medium in duplicate. Plates were incubated at 28 °C for 4 d. To visualize the hydrolysis zone, the plates were flooded with an aqueous solution of 0.1% Congo red for 15 min and washed with 1 M NaCl [79]. The experiment was repeated three times independently.

#### 4.4.9. Pectinase Activity

Ammonium mineral agar (AMA) plates were used. Plates were revealed with 2% (*w*/*v*) CTAB to show a halo around positive bacteria [79].

### 4.5. Tolerance of Isolates to Acid and Alkaline pH, Temperature, Sodium Chloride and Heavy Metals

For pH and salt tolerance characterization, the growth of isolates was observed at 28 °C for 3 days in TY plates supplemented with 0.5–8% NaCl [80] or adjusted within a pH range of 4–12. The bacteria were cultivated on TY plates, exposing them to ascending temperatures spanning from 25 to 40 °C, over a period of 4 days.

Bacteria were plated in TY medium with increasing concentrations (25 µM to 2000 µM) of Pb, Cd, Cr, Cu, Ni and Zn, from 500 mM NiCl_2_, PbCl_2_, CdCl_2_, K_2_Cr_2_O_7_, CuCl_2_ and ZnCl_2_ stock solutions. Plates were incubated for 3 days at 28 °C. Tolerance or visible bacterial growth in the maximum concentration was expressed as the maximum tolerable concentration (MTC). The sensitivity of the tested isolates was determined by assessing growth inhibition on TY agar plates that were drop-inoculated with 2 μL of fresh exponentially growing cultures [66]. Colony growth was assessed in triplicate plates from two technical duplicates.

### 4.6. Lupin Seed Germination Assay

White lupin (*L. albus* var. Orden Dorado) seeds were surface-sterilized with 70% ethanol for 1 min and treated with 25% sodium hypochlorite for 5 min followed by six washes with sterile distilled water. Surface sterilized seeds were primed by immersion into the corresponding bacterial inoculum for 1 h under shaking. Control seeds were immersed in sterile medium. Seeds were germinated on sterile Petri dishes containing filter paper wetted with 50 mL of each saline concentration (0, 60 and 100 mM). Ten seeds were geminated in triplicate for each concentration. Subsequently, batches of 30 seeds inoculated with bacteria were positioned on 0.9% (*w*/*v*) agar in Petri dishes, with 10 seeds per plate in triplicate for each treatment. These plates were then kept in darkness at 28 °C for a duration of 7 days. The percentage of emergence was calculated with the following formula:

$$\mathrm{Emergence}\;\%=\frac{\mathrm{Number}\;\mathrm{of}\;\mathrm{emerging}\;\mathrm{seedlings}}{\mathrm{Number}}\times 100$$

### 4.7. Seed Inoculation and Plant Growth

The plant growth assay was performed as previously described [81] in an environmentally controlled growth chamber. Two PGP isolates, L1 and L12, were tested for their effects on the growth parameters. These two strains were tested individually and in consortium with *Bradyrhizobium canariense* L-7Q. Bacterial strains were grown in TY medium. Exponentially growing cells in shaken broth culture were inoculated. Lupin seeds were sterilized according to the protocol described above. After that, seeds were soaked in the different bacterial suspensions. Inoculation treatments were (1) Control (not inoculated), (2) L1 (L1 primed seeds), (3) L12 (L12 primed seeds), (4) L-7Q (L-7Q primed seeds), (5) L1 + L-7Q, and (6) L12 + L-7Q. Six germinated seeds were inoculated, and placed in sterilized 1 L plastic containers filled with vermiculite (previously washed and decanted). The plants were watered three times per week. The first week with sterile distilled water. The second week with 1/4 diluted nitrogen-free nutrient solution [82]. The third week with 1/2 diluted solution, and from the fourth week on with non-diluted solution until plants were harvested. The plants were grown in a growth chamber under a 14 h light (23 °C)/10 h dark (20 °C) photoperiod for six weeks.

Six-week-old plants were harvested (six plants per treatment) and analyzed for growth-related parameters, including leaf area and root/shoot fresh and dry weight. Roots and shoots were oven-dried separately at 60 °C for 3 days to determine dry weight. For leaf area, two of the youngest fully expanded leaves per plant, i.e., 12 leaves per treatment, were scanned using an Epson Perfection V350 Photo scanner (Suwa, Japan). Images were processed using the ImageJ v.1.54 software (NIH, Bethesda, Rockville, MD, USA) to obtain leaf areas.

### 4.8. Statistical Analyses

Data were analyzed with the SPSS v27.0 software package (IBM SPSS Inc., Chicago, IL, USA). The results of each treatment/inoculation were compared using one-way ANOVA. Significant differences among treatments were separated by the least significant difference test or the Tukey B test (*p* ≤ 0.05).

## Figures and Tables

**Table 1:** PGP traits and enzymatic activities of lupin nodule-associated bacterial strains isolated from Tunisian soil.

Strain	Nitrogen Fixation	Phosphate Solubilization	IAA Production *	ACC Deaminase Activity **	Siderophore Production	Enzyme Activities		
						Lipase	Cellulase	Pectinase
L1	+	++	8.93	22.02 ± 0.56	++	+	+	-
L4	-	-	1.65	-	-	+	-	-
L5	-	±	-	-	-	+	-	-
L6	±	-	-	-	±	+	-	-
L9	-	-	-	-	-	-	-	-
L12	+	++	8.89	22.16 ± 0.29	++	+	+	-
L13	+	+	-	-	+	+	-	-
L15	+	+	12.32	-	+	+	-	-
L18	+	-	8.45	-	-	-	+	-
L19	±	-	8.95	-	-	-	-	-
L21	+	-	4.82	-	-	-	+	-

-, no growth/activity; ±, weak growth/activity (10–30% compared to the control in TY); +, good growth/activity (similar to the control); ++, very good growth/activity. * mg L^−1^, ** μmol α-ketobutyrate mg protein^−1^ h^−1^.

**Table 2:** pH and temperature growth range and the tolerance to NaCl and heavy metals. Tolerance values are shown as maximum tolerable concentrations for NaCl (mM) and heavy metals (μM).

Strain	pH	Temperature (°C)	NaCl MTC (mM)	Heavy Metals MTC (μM)					
				Zn	Cd	Cr	Pb	Ni	Cu
L1	4–12	25–40	850	750	200	150	1750	2000	2000
L4	4–12	25–40	340	750	50	450	2000	1000	2000
L5	4–12	25–40	1200	500	75	450	2000	1000	2000
L6	4–12	25–40	1200	500	25	250	2000	1500	1500
L9	7–9	25–40	170	150	00	100	1750	1000	1500
L12	4–12	25–40	850	750	200	150	2000	1500	2000
L13	4–12	25–40	1200	750	100	450	2000	2000	2000
L15	4–12	25–40	850	750	500	150	1750	2000	2000
L18	4–10	25–40	340	750	150	50	2000	1000	2000
L19	4–12	25–40	340	750	100	100	1750	1000	1000
L21	4–10	25–40	340	750	150	150	2000	1000	2000

## Data Availability

The data presented in this study are available on request from the corresponding author. Original data collected and processed to obtain the results presented in this study are not publicly available due to their lack of interest.

## References

[1] Lucas M.M., Stoddard F.L., Annicchiarico P., Frías J., Martínez-Villaluenga C., Sussmann D., Duranti M., Seger A., Zander P.M., Pueyo J.J. (2015). The future of lupin as a protein crop in Europe. Front. Plant Sci..

[2] Leporatti M.L., Ghedira K. (2009). Comparative analysis of medicinal plants used in traditional medicine in Italy and Tunisia. J. Ethnobiol. Ethnomed..

[3] Ishaq A.R., El-Nashar H.A.S., Younis T., Mangat M.A., Shahzadi M., Ul Haq A.S., El-Shazly M. (2022). Genus *Lupinus* (Fabaceae): A review of ethnobotanical, phytochemical and biological studies. J. Pharm. Pharmacol..

[4] Siger A., Czubinski J., Kachlicki P., Dwiecki K., Lampart-Szczapa E., Nogala-Kalucka M. (2012). Antioxidant activity and phenolic content in three lupin species. J. Food Compos. Anal..

[5] Quiñones M.A., Fajardo S., Fernández-Pascual M., Lucas M.M., Pueyo J.J. (2021). Nodulated white lupin plants growing in contaminated soils accumulate unusually high mercury concentrations in their nodules, roots and especially cluster roots. Horticulturae.

[6] Quiñones M.A., Ruiz-Díez B., Fajardo S., López-Berdonces M.A., Higueras P.L., Fernández-Pascual M. (2013). *Lupinus albus* plants acquire mercury tolerance when inoculated with an Hg-resistant *Bradyrhizobium* strain. Plant Physiol. Biochem..

[7] Pueyo J.J., Quiñones M.A., Coba de la Peña T., Fedorova E.E., Lucas M.M. (2021). Nitrogen and phosphorus interplay in lupin root nodules and cluster roots. Front. Plant Sci..

[8] Quiñones M.A., Lucas M.M., Pueyo J.J. (2022). Adaptive mechanisms make lupin a choice crop for acidic soils affected by aluminum toxicity. Front. Plant Sci..

[9] Msaddak A., Mars M., Quiñones M.A., Lucas M.M., Pueyo J.J. (2023). Lupin, a unique legume that is nodulated by multiple microsymbionts: The role of horizontal gene transfer. Int. J. Mol. Sci..

[10] Poole P., Ramachandran V., Terpolilli J. (2018). Rhizobia: From saprophytes to endosymbionts. Nat. Rev. Microbiol..

[11] Rajendran G., Patel M.H., Joshi S.J. (2012). Isolation and characterization of nodule-associated *Exiguobacterium* sp. from the root nodules of *Fenugreek* (*Trigonella foenum-graecum*) and their possible role in plant growth promotion. Int. J. Microbiol..

[12] De Meyer S.E., De Beuf K., Vekeman B., Willems A. (2015). A large diversity of non-rhizobial endophytes found in legume root nodules in Flanders (Belgium). Soil Biol. Biochem..

[13] Velázquez E., Valverde A., Rivas R., Gomis V., Peix Á., Gantois I., Igual J.M., León-Barrios M., Willems A., Mateos P.F. (2010). Strains nodulating *Lupinus albus* on different continents belong to several new chromosomal and symbiotic lineages within *Bradyrhizobium*. Anton. Leeuw..

[14] Kloepper J.W., Schroth M.N. Plant growth-promoting rhizobacteria on radishes. Proceedings of the IVth International Conference on Plant Pathogenic Bacteria Volume 2, Station de Pathologie Vegetale et Phyto-Bacteriologie.

[15] Pinter I.F., Salomon M.V., Berli F., Bottini R., Piccoli P. (2017). Characterization of the As(III) tolerance conferred by plant growth promoting rhizobacteria to in vitro-grown grapevine. Appl. Soil Ecol..

[16] Pucciariello C., Boscari A., Tagliani A., Brouquisse R., Perata P. (2019). Exploring legume-rhizobia symbiotic models for waterlogging tolerance. Front. Plant Sci..

[17] Chamkhi I., El Omari N., Balahbib A., El Menyiy N., Benali T., Ghoulam C. (2022). Is the rhizosphere a source of applicable multi-beneficial microorganisms for plant enhancement?. Saudi J. Biol. Sci..

[18] Roy S., Liu W., Nandety R.S., Crook A., Mysore K.S., Pislariu C.I., Frugoli J., Dickstein R., Udvardi M.K. (2020). Celebrating 20 years of genetic discoveries in legume nodulation and symbiotic nitrogen fixation. Plant Cell.

[19] Etesami H., Jeong B.R., Glick B.R. (2021). Contribution of arbuscular mycorrhizal fungi, phosphate–solubilizing bacteria, and silicon to P uptake by plant. Front. Plant Sci..

[20] Lurthy T., Cantat C., Jeudy C., Declerck P., Gallardo K., Barraud C., Leroy F., Ourry A., Lemanceau P., Salon C. (2020). Impact of bacterial siderophores on iron status and ionome in pea. Front. Plant Sci..

[21] Kang S.-M., Shahzad R., Khan M.A., Hasnain Z., Lee K.-E., Park H.-S., Kim L.-R., Lee I.-J. (2021). Ameliorative effect of indole-3-acetic acid- and siderophore-producing *Leclercia adecarboxylata* MO1 on cucumber plants under zinc stress. J. Plant Interact..

[22] Raza A., Razzaq A., Mehmood S., Zou X., Zhang X., Lv Y., Xu J. (2019). Impact of climate change on crops adaptation and strategies to tackle its outcome: A review. Plants.

[23] Navarro-Torre S., Bessadok K.J., Flores-Duarte N.D., Rodríguez-Llorente I., Caviedes M.A., Pajuelo E. (2020). Helping legumes under stress situations: Inoculation with beneficial microorganisms. Legume Crops.

[24] Penrose D.M., Glick B.R. (2003). Methods for isolating and characterizing ACC deaminase-containing plant growth-promoting rhizobacteria. Physiol. Plant..

[25] Chandwani S., Amaresan N. (2022). Role of ACC deaminase producing bacteria for abiotic stress management and sustainable agriculture production. Environ. Sci. Pollut. Res..

[26] Alemneh A.A., Zhou Y., Ryder M.H., Denton M.D. (2020). Mechanisms in plant growth-promoting rhizobacteria that enhance legume–rhizobial symbioses. J. Appl. Microbiol..

[27] Shiraishi A., Matsushita N., Hougetsu T. (2010). Nodulation in black locust by the gammaproteobacteria *Pseudomonas* sp. and the betaproteobacteria *Burkholderia* sp.. Syst. Appl. Microbiol..

[28] Martínez-Hidalgo P., Hirsch A.M. (2017). The Nodule Microbiome: N_2_-fixing rhizobia do not live alone. Phytobiomes J..

[29] Bessadok K., Navarro-Torre S., Pajuelo E., Mateos-Naranjo E., Redondo-Gómez S., Caviedes M.Á., Fterich A., Mars M., Rodríguez-Llorente I.D. (2020). The ACC-deaminase producing bacterium *Variovorax* sp. CT7.15 as a tool for improving *Calicotome villosa* nodulation and growth in arid regions of Tunisia. Microorganisms.

[30] Peix A., Ramírez-Bahena M.H., Velázquez E., Bedmar E.J. (2015). Bacterial associations with legumes. Crit. Rev. Plant Sci..

[31] Ferchichi N., Toukabri W., Boularess M., Smaoui A., Mhamdi R., Trabelsi D. (2019). Isolation, identification and plant growth promotion ability of endophytic bacteria associated with lupine root nodule grown in Tunisian soil. Arch. Microbiol..

[32] Tounsi-Hammami S., Le Roux C., Dhane-Fitouri S., De Lajudie P., Duponnois R., Ben Jeddi F. (2019). Genetic diversity of rhizobia associated with root nodules of white lupin (*Lupinus albus* L.) in Tunisian calcareous soils. Syst. Appl. Microbiol..

[33] Mahanty T., Bhattacharjee S., Goswami M., Bhattacharyya P., Das B., Ghosh A., Tribedi P. (2017). Biofertilizers: A potential approach for sustainable agriculture development. Environ. Sci. Pollut. Res..

[34] Nieto J.J., Fernández-Castillo R., Márquez M.C., Ventosa A., Quesada E., Ruiz-Berraquero F. (1989). Survey of metal tolerance in moderately halophilic eubacteria. Appl. Environ. Microbiol..

[35] Coutinho H.L.C., Oliveira V.M., Lovato A., Maia A.H.N., Manfio G.P. (1999). Evaluation of the diversity of rhizobia in Brazilian agricultural soils cultivated with soybeans. Appl. Soil Ecol..

[36] Durán D., Rey L., Sánchez-Cañizares C., Navarro A., Imperial J., Ruiz-Argueso T. (2013). Genetic diversity of indigenous rhizobial symbionts of the *Lupinus mariae-josephae* endemism from alkaline-limed soils within its area of distribution in Eastern Spain. Syst. Appl. Microbiol..

[37] McInnes A. (2004). Structure and diversity among rhizobial strains, populations and communities? A review. Soil Biol. Biochem..

[38] Rivas R., Peix A., Mateos P.F., Trujillo M.E., Martínez-Molina E., Velázquez E. (2006). Biodiversity of populations of phosphate solubilizing rhizobia that nodulate chickpea in different Spanish soils. Plant Soil.

[39] Young J.M., Kuykendall L.D., Martínez-Romero E., Kerr A., Sawada H. (2001). A revision of *Rhizobium* Frank 1889, with an emended description of the genus, and the inclusion of all species of *Agrobacterium* Conn 1942 and *Allorhizobium undicola* de Lajudie et al. 1998 as new combinations: *Rhizobium radiobacter*, *R. rhizogenes*, *R. rubi*, *R. undicola* and *R. vitis*. Int. J. Syst. Evol. Microbiol..

[40] Zahradník J., Nunvar J., Pařízková H., Kolářová L., Palyzová A., Marešová H., Grulich M., Kyslíková E., Kyslík P. (2018). *Agrobacterium bohemicum* sp. nov. isolated from poppy seed wastes in Central Bohemia. Syst. Appl. Microbiol..

[41] Aserse A.A., Räsänen L.A., Assefa F., Hailemariam A., Lindström K. (2012). Phylogeny and genetic diversity of native rhizobia nodulating common bean (*Phaseolus vulgaris* L.) in Ethiopia. Syst. Appl. Microbiol..

[42] Li Y., Li X., Liu Y., Wang E.T., Ren C., Liu W., Xu H., Wu H., Jiang N., Li Y. (2016). Genetic diversity and community structure of rhizobia nodulating *Sesbania cannabina* in saline–alkaline soils. Syst. Appl. Microbiol..

[43] Valdez-Nuñez R.A., Castro-Tuanama R., Castellano-Hinojosa A., Bedmar E.J., Ríos-Ruiz W.F., Zúñiga-Dávila D., González-Andrés F., Ormeño-Orrillo E. (2019). PGPR characterization of non-nodulating bacterial endophytes from root nodules of *Vigna unguiculata* (L.) Walp. Microbial Probiotics for Agricultural Systems.

[44] Waraczewska Z., Niewiadomska A., Wolna-Maruwka A., Sulewska H., Budka A., Pilarska A.A. (2022). The effect of in vitro coinoculation on the physiological parameters of white lupine plants (*Lupinus albus* L.). Appl. Sci..

[45] Zakhia F., Jeder H., Willems A., Gillis M., Dreyfus B., De Lajudie P. (2006). Diverse bacteria associated with root nodules of spontaneous legumes in Tunisia and first report for *nifH*-like gene within the genera *Microbacterium* and *Starkeya*. Microb. Ecol..

[46] Deng Z.S., Zhao L.F., Kong Z.Y., Yang W.Q., Lindström K., Wang E.T., Wei G.H. (2011). Diversity of endophytic bacteria within nodules of the *Sphaerophysa salsula* in different regions of Loess Plateau in China. FEMS Microbiol. Ecol..

[47] Leite H.A.C., Silva A.B., Gomes F.P., Gramacho K.P., Faria J.C., de Souza J.T., Loguercio L.L. (2013). *Bacillus subtilis* and *Enterobacter cloacae* endophytes from healthy *Theobroma cacao* L. trees can systemically colonize seedlings and promote growth. Appl. Microbiol. Biotechnol..

[48] Kilian M., Steiner U., Krebs B., Junge H., Schmiedeknecht G., Hain R. (2000). FZB24^®^
*Bacillus subtilis*—Mode of action of a microbial agent enhancing pant vitality. Pflanzenschutz Nachr. Bayer.

[49] Tsavkelova E.A., Cherdyntseva T.A., Klimova S.Y., Shestakov A.I., Botina S.G., Netrusov A.I. (2007). Orchid-associated bacteria produce indole-3-acetic acid, promote seed germination, and increase their microbial yield in response to exogenous auxin. Arch. Microbiol..

[50] Martínez-Viveros O., Jorquera M.A., Crowley D.E., Gajardo G., Mora M.L. (2010). Mechanisms and practical considerations involved in plant growth promotion by rhizobacteria. J. Soil Sci. Plant Nutr..

[51] Miransari M., Smith D.L. (2014). Plant hormones and seed germination. Environ. Exp. Bot..

[52] Bashan Y., de-Bashan L.E., Cassán F.D., Okon Y., Creus C.M. (2015). Inoculant preparation and formulations for *Azospirillum* spp.. Handbook for Azospirillum.

[53] Ahmad F., Ahmad I., Khan M.S. (2008). Screening of free-living rhizospheric bacteria for their multiple plant growth promoting activities. Microbiol. Res..

[54] Panwar M., Tewari R., Nayyar H., Khan M., Zaidi A., Musarrat J. (2014). Microbial consortium of plant growth-promoting rhizobacteria improves the performance of plants growing in stressed soils: An overview. Phosphate Solubilizing Microorganisms.

[55] Jha C.K., Saraf M. (2015). Plant growth promoting rhizobacteria (PGPR): A review. J. Agric. Res. Dev..

[56] Babalola O.O. (2010). Beneficial bacteria of agricultural importance. Biotechnol. Lett..

[57] Zhang J., Wang P., Fang L., Zhang Q.-A., Yan C., Chen J. (2017). Isolation and characterization of phosphate-solubilizing bacteria from mushroom residues and their effect on tomato plant growth promotion. Pol. J. Microbiol..

[58] Patel P., Trivedi G., Saraf M. (2018). Iron biofortification in mungbean using siderophore producing plant growth promoting bacteria. Environ. Sust..

[59] Turan M., Kitir N., Alkaya Ü., Günes A., Tüfenkçi S., Yildirim E., Nikerel E., Rigobelo E.C. (2016). Making soil more accessible to plants: The case of plant growth promoting rhizobacteria. Plant Growth.

[60] Gupta G., Parihar S.S., Ahirwar N.K., Snehi N.K. (2015). Plant growth promoting rhizobacteria (PGPR): Current and future prospects for development of sustainable agriculture. J. Microb. Biochem. Technol..

[61] Selim S.M., Zayed M.S., Panpatte D.G., Jhala Y.K., Vyas R.V., Shelat H.N. (2017). Role of biofertilizers in sustainable agriculture under abiotic stresses. Microorganisms for Green Revolution.

[62] Kang B.G., Kim W.T., Yun H.S., Chang S.C. (2010). Use of plant growth-promoting rhizobacteria to control stress responses of plant roots. Plant Biotechnol. Rep..

[63] Menéndez E., Robledo M., Jiménez-Zurdo J.I., Velázquez E., Rivas R., Murray J.D., Mateos P.F. (2019). Legumes display common and host-specific responses to the rhizobial cellulase CelC_2_ during primary symbiotic infection. Sci. Rep..

[64] Mwenda G.M., Hill Y.J., O’Hara G.W., Reeve W.G., Howieson J.G., Terpolilli J.J. (2023). Competition in the *Phaseolus vulgaris*-*Rhizobium* symbiosis and the role of resident soil rhizobia in determining the outcomes of inoculation. Plant Soil.

[65] Msaddak A., Rey L., Imperial J., Palacios J.M., Mars M., Pueyo J.J. (2021). Phylogenetic analyses of rhizobia isolated from nodules of *Lupinus angustifolius* in Northern Tunisia reveal *Devosia* sp. as a new microsymbiont of lupin species. Agronomy.

[66] Nonnoi F., Chinnaswamy A., García de la Torre V.S., Coba de la Peña T., Lucas M.M., Pueyo J.J. (2012). Metal tolerance of rhizobial strains isolated from nodules of herbaceous legumes (*Medicago* spp. and *Trifolium* spp.) growing in mercury-contaminated soils. Appl. Soil Ecol..

[67] Sanger F., Nicklen S., Coulson A.R. (1977). DNA sequencing with chain-terminating inhibitors. Proc. Natl. Acad. Sci. USA.

[68] Yoon S.-H., Ha S.-M., Kwon S., Lim J., Kim Y., Seo H., Chun J. (2017). Introducing EzBioCloud: A taxonomically united database of 16S rRNA gene sequences and whole-genome assemblies. Int. J. Syst. Evol. Microbiol..

[69] Chenna R. (2003). Multiple sequence alignment with the Clustal series of programs. Nucleic Acids Res..

[70] Kumar S., Stecher G., Tamura K. (2016). MEGA7: Molecular evolutionary genetics analysis version 7.0 for bigger datasets. Mol. Biol. Evol..

[71] Kirchhof G., Reis V.M., Baldani J.I., Eckert B., Dobereiner J., Hartmann A. (1997). Occurrence, physiological and molecular analysis of endophytic diazotrophic bacteria in gramineous energy plants. Plant Soil.

[72] Nautiyal C.S. (1999). An efficient microbiological growth medium for screening phosphate solubilizing microorganisms. FEMS Microbiol. Lett..

[73] Glickmann E., Dessaux Y. (1995). A critical examination of the specificity of the Salkowski reagent for indolic compounds produced by phytopathogenic bacteria. Appl. Environ. Microbiol..

[74] Bradford M.M. (1976). A rapid and sensitive method for the quantitation of microgram quantities of protein utilizing the principle of protein-dye binding. Anal. Biochem..

[75] Honma M., Shimomura T. (1978). Metabolism of 1-aminocyclopropane-1-carboxylic acid. Agric. Biol. Chem..

[76] Alexander D.B., Zuberer D.A. (1991). Use of chrome azurol S reagents to evaluate siderophore production by rhizosphere bacteria. Biol. Fertil. Soils.

[77] Fasim F., Ahmed N., Parsons R., Gadd G.M. (2002). Solubilization of zinc salts by a bacterium isolated from the air environment of a tannery. FEMS Microbiol. Lett..

[78] Sharma S.K. (2012). Characterization of zinc-solubilizing *Bacillus* isolates and their potential to influence zinc assimilation in soybean seeds. J. Microbiol. Biotechnol..

[79] Elbeltagy A., Nishioka K., Suzuki H., Sato T., Sato Y.-I., Morisaki H., Mitsui H., Minamisawa K. (2000). Isolation and characterization of endophytic bacteria from wild and traditionally cultivated rice varieties. Soil Sci. Plant Nutr..

[80] Barra P.J., Inostroza N.G., Acuña J.J., Mora M.L., Crowley D.E., Jorquera M.A. (2016). Formulation of bacterial consortia from avocado (*Persea americana* Mill.) and their effect on growth, biomass and superoxide dismutase activity of wheat seedlings under salt stress. Appl. Soil Ecol..

[81] Penrose D.M., Moffatt B.A., Glick B.R. (2001). Determination of 1-aminocycopropane-1-carboxylic acid (ACC) to assess the effects of ACC deaminase-containing bacteria on roots of canola seedlings. Can. J. Microbiol..

[82] Hoagland D.R., Arnon D.I. (1938). The Water-Culture Method for Growing Plants without Soil.

